# Unlocking the Benefits of Hybrid and Standalone Pervaporation for Sustainable Isopropanol Dehydration with HybSi^®^ AR Membranes

**DOI:** 10.3390/membranes15080224

**Published:** 2025-07-26

**Authors:** Mohammed Nazeer Khan, Elmar Boorsma, Pieter Vandezande, Ilse Lammerink, Rob de Lange, Anita Buekenhoudt, Miet Van Dael

**Affiliations:** 1 Unit Materials & Chemistry (MatCh), Flemish Institute for Technological Research (VITO), Boeretang 200, 2400 Mol, Belgium; anita.buekenhoudt@vito.be (A.B.); miet.vandael@vito.be (M.V.D.); 2 Pervatech B.V, 7463 Rijssen, The Netherlandsilse.lammerrink@pervatech.nl (I.L.); ron.delange@aswyn.nl (R.d.L.); 3 Aswyn, Engelse Schans 12, 7137 SE Lievelde, The Netherlands; 4 Centre for Environmental Sciences (CMK), Hasselt University, Agoralaan, 3590 Diepenbeek, Belgium

**Keywords:** pervaporation, techno-economic assessment, solvent recovery, solvent dehydration, CO_2_ reduction, azeotropic distillation

## Abstract

This study presents the first combined techno-economic and environmental analysis of IPA dehydration using HybSi^®^ membranes across three configurations, offering a low-emission alternative to conventional azeotropic distillation. The processes are simulated in Aspen Plus, and include two hybrid separation processes (i.e., distillation–pervaporation and distillation–pervaporation–distillation) and one standalone pervaporation process. The pervaporation module uses data from experiments that were performed using HybSi^®^ AR membranes at 130 °C and two vacuum pressures (20 and 50 mbar). The separation processes were systematically compared using a comprehensive set of performance indicators covering technical, economic, and environmental aspects. A new cost-efficiency metric, *COPCO*, is introduced, alongside updated modeling under 2024 market conditions. The isopropanol recovery and water selectivity were >99.5% and >98.7%, respectively, in all pervaporation-based processes. It was found that the hybrid distillation–pervaporation process resulted in a 42% reduction in the levelized cost of the benchmark azeotropic distillation process, while standalone pervaporation resulted in a 38% reduction. The CO_2_ footprint was also reduced significantly in all cases, up to 86% in the case of standalone pervaporation compared to azeotropic distillation. The *COPCO* analysis revealed that the distillation–pervaporation configuration offers the highest cost-efficiency among the evaluated systems. Sensitivity analysis revealed that feed flow rate, average water flux, membrane module price, membrane lifetime, and steam price significantly impact the levelized cost. Lower vacuum pressure and feed water near the azeotropic composition enhance economic performance.

## 1. Introduction

The recovery and reuse of solvents are important objectives in many industries to improve sustainability and circularity, as well as to reduce operating costs and carbon footprint. In many applications, water is typically the primary contaminant that must be removed before the solvent can be reused [1]. The chemical industry relies extensively on thermal-based separation processes, such as distillation, to dehydrate the solvent and achieve high purity. These separation processes are energy-intensive, primarily utilizing steam generated from the combustion of natural gas. In recent years, the cost of steam has escalated significantly, with further increases anticipated due to diminishing natural resource supplies and the 2050 climate goals [2]. The pressure to reduce energy consumption and CO_2_ emissions triggers companies to explore alternative separation methods, such as membrane technologies or hybrid systems. One notable example of such a hybrid system is combining pervaporation with distillation. The use of a simple distillation process to complete purification is not possible or is energy- and capital-intensive when dealing with azeotropes or close boiling points. In such cases, a hybrid process combining distillation and pervaporation, or a standalone pervaporation process, can be an attractive alternative.

The market for pervaporation membranes is expected to witness significant growth in the coming years, primarily driven by the growing demand for solvent recovery, comprising dehydration as well as the separation of organic solvents. On average, only 39% of spent solvents were recycled in the EU-28 in 2018 [3]. The current market for pervaporation membranes was valued at 4.8 billion euros in 2023 and is projected to reach 6.7 billion euros, growing at a compound annual growth rate (CAGR) of 6.9% during the forecasted period 2024 to 2031 [4]. Pervatech developed and commercialized a unique and robust hybrid silica membrane (HybSi^®^ AR), designed to separate water or other small polar compounds from solvents. They have superior acid resistance and are a good fit for a variety of dehydrations and throughputs [5]. A full-scale isopropanol (IPA) dehydration plant using HybSi^®^ AR membranes in vapor permeation mode was already realized by Pervatech [5]. These membranes are being used in a wide variety of industrial applications such as the dehydration of both process and waste solvent mixtures relevant to chemical, pharma, food, and biotech processing. Advantages of HybSi^®^ AR membranes include superior chemical and thermal stability, and up to 50% potential savings in energy. The reader can refer to the suggested literature for more information on general solvent recovery [6], dehydration by pervaporation [1], pervaporation membrane materials [7], and specifically ceramic PV membrane materials [8,9].

Isopropyl alcohol (IPA) has numerous applications, including use as a solvent, cleaning agent, and disinfectant. The majority of its market share comes from its role as a solvent in various industries, such as pharmaceuticals, chemicals, and electronics [10]. The global market volume of isopropyl alcohol was approximately 2.3 million metric tons. Europe is the third-largest market for IPA, largely due to the presence of several prominent pharmaceutical companies [10].

The separation of IPA from water, particularly near its azeotropic composition (~87 wt.% IPA), presents both thermodynamic and economic challenges. Traditional azeotropic distillation, though widely used, is energy-intensive and costly when using entrainers like benzene [11]. Hybrid distillation–pervaporation (D–PV) processes, especially those employing NaA-type ceramic membranes, have shown significantly improved economics. It was reported that incorporating pervaporation reduced the total separation cost by 49%, while maintaining a product purity of 99.5 wt.% IPA [11]. Membrane performance data support these gains, as zeolite NaA membranes demonstrated water/IPA separation factors of ~10,000 and fluxes up to 1.76–2.0 kg/m^2^·h at 70–90 °C [9]. Further enhancement was observed in vapor permeation–distillation (D–VP) hybrids, where Harvianto et al. reported reductions in separation cost by 77% compared to azeotropic distillation, largely due to a lower energy input and the elimination of entrainer handling [12]. These systems delivered comparable purities (>99.5 wt.% IPA) and benefited from the high thermal stability and selectivity of hollow fibre modules. Furthermore, Spatolisano and Pellegrini reviewed process intensification strategies and proposed alternative schemes combining azeotropic distillation and extraction, confirming energy savings over traditional setups, though without matching the cost-effectiveness of membrane-based routes [13]. Overall, techno-economic assessments favour membrane-assisted processes, particularly those using ceramic materials, for their ability to combine high separation efficiency, lower energy demands, and favorable operational costs, making them strong candidates for sustainable IPA recovery at scale.

Recent studies have showcased various advancements in membrane design and modeling for IPA–water dehydration. Flexible hybrid silica membranes fabricated via ultrasonic spray coating achieved a water flux of approximately 0.6 kg/(m^2^·h) and a separation factor of approximately 1300 for 90 wt.% IPA solutions, demonstrating scalability and high performance [14]. Vacuum-assisted interfacial polymerization produced thin-film composite polyamide membranes with a water flux of approximately 1.504 kg/(m^2^·h) and a separation factor of approximately 314 for 70 wt.% IPA feeds [15]. Meanwhile, mixed-matrix membranes incorporating In (BTC) MOFs into PVA reported fluxes of 0.142–0.341 kg/(m^2^·h) with permeate water purities up to 99.9 wt.% [16], and PVA membranes enhanced with kaolinite showed a flux of 0.86 kg/(m^2^·h) in ternary solvent systems (epichlorohydrin/IPA/water) [17].

Despite these recent material-level developments, relatively few studies integrate detailed membrane performance into system-level techno-economic analysis (TEA). Aziaba et al. (2022) developed a modular solution–diffusion-based pervaporation model compatible with open-source simulators and validated against experimental results [18]. Separately, Van Hecke et al. (2021) embedded experimental HybSi^®^ membrane performance data into a digital twin simulation for the intensification of transesterification processes, enabling process optimization and membrane sizing via TEA principles [19]. These efforts illustrate the growing importance of combining experimental data with economic and environmental metrics, a direction further explored in the present study.

Several modeling studies and economic assessments of IPA dehydration are available in the literature. One such notable study, conducted about two decades ago, was reported by Van Hoof et al. [11]. In this study, economic evaluations were carried out based on experimental data obtained with two commercial membranes available at the time, i.e., a polymeric membrane (i.e., PERVAP^®^ 2510, Sulzer Chemtech, Germany) and one ceramic membrane (i.e., NaA zeolite, Mitsui & Co.). These membranes have limited selectivity and permeability compared to current HybSi^®^ AR membranes that combine the best properties of organic and inorganic materials. For example, the Mitsui membrane had a flux of approximately 4 kg/h∙m^2^ at a feed water concentration of 10 wt.% [11] as opposed to the HybSi^®^ AR membrane in this study, which showed a flux of nearly 20 kg/h∙m^2^ under similar conditions. This paper has been and is being highly cited, and is considered very relevant by the industry. However, the data used in the paper on energy and membrane costs, as well as membrane performance, are outdated.

This study presents the first combined techno-economic and environmental analysis of IPA dehydration using HybSi^®^ membranes across three configurations. A new cost efficiency metric, *COPCO*, is introduced, alongside updated modeling under 2024 market conditions. The results in terms of technical, economic, and environmental performance were compared with a benchmark azeotropic distillation process. This was achieved by conducting experimental pervaporation experiments, as well as simulations and model calculations of distillation columns. Compared to the study by Van Hoof et al. [11], important additional costs such as the outside battery limit (OSBL) costs, insurance, labor, and waste disposal costs were also considered. This study aims to inform a broad audience on the technical capabilities, energy, and separation costs of pervaporation technology for solvent recovery, more specifically for the dehydration of a highly relevant solvent/water system, i.e., IPA-water. By quantifying the energy/cost gains, CO_2_ footprint reduction, and process intensification potential, industrial implementation of this alternative technology, as well as market uptake of HybSi^®^ membranes, can be further pushed. To further evaluate the techno-economic potential of pervaporation for solvent recovery, the insights and methodology adopted in this study will be extended to several other industrially relevant solvent/water mixtures in a future paper.

## 2. Materials and Methods

The following sections present the details of the pervaporation experiments with the IPA-H_2_O system and process modeling of the benchmark azeotropic distillation (D-D-D), and three pervaporation-based processes. Of these latter three processes, two are hybrid cases (distillation–pervaporation, D-PV, and distillation–pervaporation–distillation, D-PV-D), and the other is a standalone pervaporation (PV).

### 2.1. Pervaporation Experiments

**Figure 1:**
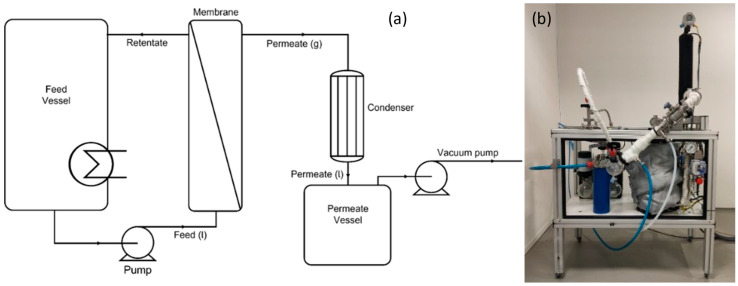
(**a**) Schematic overview of the lab-scale pervaporation equipment. (**b**) Photograph of the experimental laboratory apparatus.

A schematic overview of the equipment used for the pervaporation experiments and a photograph of the experimental laboratory apparatus are shown in Figure 1. Experiments were performed with a set of four HybSi^®^ AR membranes with a total membrane area of 0.042 m^2^ (Pervatech, The Netherlands). For each experiment, a feed mixture was prepared containing IPA (technical grade, Boom) and reverse osmosis (RO) water. The feed vessel was filled with approximately 1.5–2 kg of the prepared mixture. The mixture was then circulated over the membranes at 150 L/h and heated to a temperature of 130 °C. The feed was initially recirculated at ~1 bar, but the pressure rose to 5 bar at 130 °C during operation due to thermal expansion in the closed-loop system. A vacuum pump was used to reduce the pressure in the permeate compartment to either 20 mbar or 50 mbar. The vaporous permeate was condensed using a condenser set at a temperature of 1 °C and collected in a permeate vessel. Samples were drawn from the permeate and the retentate to monitor the pervaporation process at different time intervals while logging the feed and retentate temperatures. Sample mass was measured on a precision balance (Kern PNJ 3000-2M), while the water content was determined using coulometric Karl Fischer titration (Aquacounter AQ-300) and volumetric Karl Fischer titration (Metrohm 870 KF Titrino plus). More information on the analytical method parameters is given in Section S2 of the Supplementary Material. The water and IPA fluxes were calculated using Equation (1):

(1) $$J_{x}=\frac{m_{x}}{A\cdot t}$$

where *J_x_* is the flux (in kg m^−2^ h^−1^) of component *x* (water or IPA), and *m_x_* is the mass (in kg) of the permeant per unit of membrane area, *A* (in m^2^), and per unit of time, *t* (in h).

At the start of the pervaporation experiment, the system was unable to keep the temperature stable at 130 °C due to the high flux. In this case, water flux at 130 °C was calculated by extrapolation based on the difference in driving force determined at the experimental temperature of 130 °C using VLE data. The VLE data, along with physicochemical parameters for the IPA–water system, are given in Tables S1 and S2 of the Supplementary Material. The driving force in pervaporation is the partial pressure gradient across the membrane of the permeating component. The obtained water flux at a feed temperature of 130 °C and the appropriate vacuum pressure was monitored and plotted as a function of water content in the feed. The experimental data were fitted, and an average water flux was calculated between the initial feed and final retentate water concentration required for the modeling. The IPA flux results were from the defects present in the membrane layer. As it is not caused by a driving force across the membrane, the IPA flux cannot be fitted to the experimental data. Therefore, the maximum IPA flux was taken as the average IPA flux for modeling.

### 2.2. Process Modeling

The process modeling software Aspen Plus V14 [20] was used to model all the equipment except the vacuum pump and chiller. The pervaporation module was simulated as a separation block with specifications calculated from the experimental average flux data. The vacuum pump and chiller were not modeled explicitly, but the energy and cost data were used in the economic assessment. For distillation columns, a Rad-Frac model was used and for the heat exchangers, a heater block was used. The non-random two-liquid (NRTL) model was used to describe the liquid–vapor interactions, and is based on activity coefficients between the components [21]. The binary interaction parameters for the IPA–H_2_O system are summarized in Table S3 of the Supplementary Material and used in Aspen simulations. For all cases, a constant feed flow rate of 1000 kg/h at ambient temperature and pressure (20 °C and 1 atm) was considered. The energy (steam) consumption and cooling water requirements in distillation columns, heaters, and pumps were also estimated using Aspen Plus. To estimate the minimum number of stages, feed stage, and reflux ratio that is required to obtain the desired qualities of top and bottom streams, the design specs option and manual iteration were used wherever possible. For simplicity, the pressure drop in all equipment was neglected in this study, considering that it will have a negligible effect on overall energy consumption and costs.

#### 2.2.1. Azeotropic Distillation

**Figure 2:**
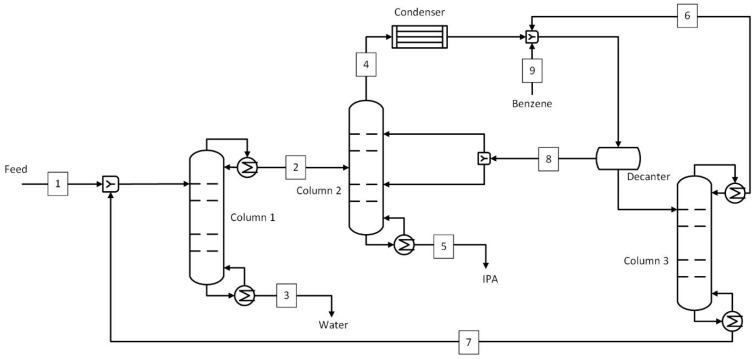
Azeotropic distillation process (benchmark). Numbered streams (1–9) represent key process flows including feed, product, and recycle streams.

An azeotropic distillation process similar to that modeled by Van Hoof et al. [11] was used as a benchmark to compare with hybrid and standalone pervaporation processes. The process flow diagram of azeotropic distillation using benzene as an entrainer is shown in Figure 2. The stream conditions corresponding to stream numbers are given in Table 1. The benchmark case was slightly modified and optimized to reduce the reflux ratio (lower reboiler heat duty and column cost) in column 3. The top stream from column 3 was mixed with the top stream from column 2 after the condenser, as opposed to before the condenser in Van Hoof et al. [11]. The feed containing a mixture of IPA and water in the ratio 50:50 wt.% was fed to column 1. This was the water removal column, which brought the top product composition (86.5 wt.% IPA and 13.5 wt.% water) close to the azeotropic composition that was fed to column 2. The bottom stream consisted mainly of water, with an IPA concentration of just 0.2 wt.%. Column 2 was the azeotropic column where benzene was added to break the azeotrope. The top stream from this column was a mixture of IPA, water, and benzene, while the bottom stream consisted of IPA at a target purity (99.5 wt.%). The mixture from the top was condensed and mixed with the top stream from column 3 and the benzene make-up, and supplied to a decanter. Here, the mixture was separated into a benzene-rich stream, which was recycled to column 2, and a water-rich stream, which was sent to column 3, a benzene recovery column. In this column, the benzene was recovered in the top stream, while the bottom stream, with a more or less similar composition as the initial feed, was sent back to the water recovery column. The number of stages, feed stage, and reflux ratios of all three columns are given in Table S9 of the Supplementary Material.

#### 2.2.2. Hybrid and Standalone Pervaporation Processes

**Figure 3:**
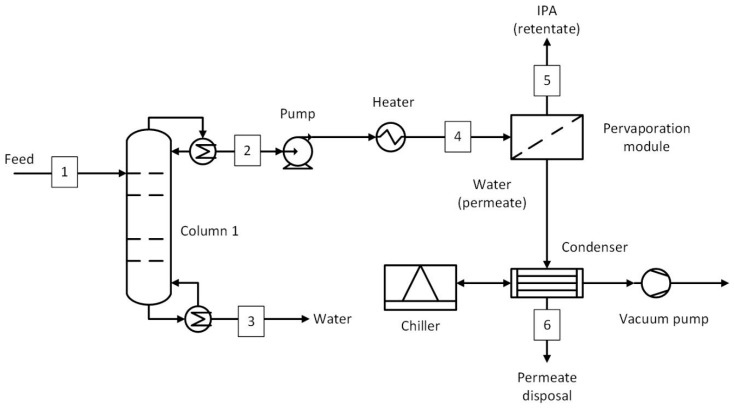
Hybrid distillation–pervaporation (D-PV) process. Numbered streams (1–6) represent key process flows including feed, product, and recycle streams.

The process flow diagram of a hybrid process involving a distillation column and pervaporation (D-PV) is shown in Figure 3, with the data for selected streams given in Table 2. Similar to the benchmark, the distillation column was used as a water removal column to bring the top stream composition close to azeotropic composition (up to 15 wt.% water), whereas the bottom stream contained mainly water with a water concentration of 99.8 wt.%. The pressure of the top stream was increased to 6 bar using a pump and then heated to a temperature of 130 °C in a heater. The selected pressure was based on VLE data and was sufficient to keep the pervaporation feed in a liquid phase. The pervaporation may require multiple modules depending on the required membrane area and area per module. The feed temperature may decrease due to evaporative cooling within the membrane modules, which decreases the pervaporation efficiency. Therefore, an interstage heater was used (not shown in the figure) to maintain the temperature at 130 °C. For simplicity, the amount of heat required was assumed to be equivalent to the heat of vaporization of water in the permeate stream. Based on the average water flux estimated from the experiments, the membrane area required was calculated. The retentate stream consists of IPA at a target purity of 99.5 wt.%, whereas the permeate stream consists of water with a concentration of 98.8 wt.%. A vacuum pump was employed to maintain a vacuum of 20 mbar on the permeate side. The flow in pervaporation systems does not contain any non-condensables in this case; therefore, a condenser before the vacuum pump was considered. The permeate was condensed at 20 mbar in a condenser with the aid of a chiller (R410A refrigerant) [22]. Using the chiller’s COP (3.4) [22] and the cooling duty requirement in the condenser (obtained from the process model), the power consumption of the chiller was calculated. The power consumption of the vacuum pump was estimated using the method defined by Seider et al. [23], while the cooling water requirement was assumed to be the same as that used in experiments per unit permeate flow rate. The flow rate to the vacuum was only the air that leaked into the system operating under vacuum. Before applying the vacuum, evacuation of the existing air may be required, but this was neglected in this study, considering the small contribution to the overall energy consumption. To estimate the air leakage rate, Equation (2), based on the equipment volume and operating pressure, was used [23]:

(2) $$W=5+\left\{0.0298+0.03088\left[ln\left(P\right)\right]-0.0005733{\left[ln\left(P\right)\right]}^{2}\right\}V^{0.66}$$

where *W* is the air leakage rate in lb/h, *P* is the operating pressure in torr, and *V* is the equipment volume in ft^3^. Here, the empty volume of the membrane module was used, which was estimated by Pervatech to be 0.03 m^3^/m^2^ area. The brake power (*B_kw_*) in kW was estimated assuming a reciprocating vacuum pump and using Equations (3) and (4), where *SF* is the size factor (lb/h/torr). Though these equations are valid for *SF* = 1.0–25, it was assumed that these are also valid for *SF* lower than 1.

(3) $$B_{kw}=3.974*{\left(SF\right)}^{0.963}$$

(4) $$SF=\frac{W}{P}$$

**Figure 4:**
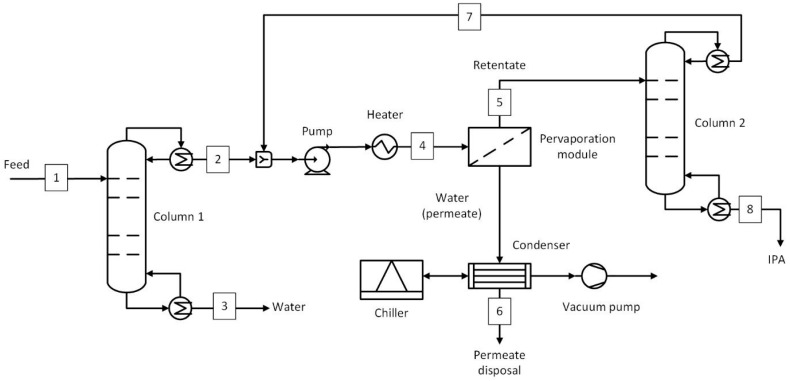
Hybrid distillation–pervaporation–distillation (D-PV-D) process. Numbered streams (1–8) represent key process flows including feed, product, and recycle streams.

The process flow diagram of the hybrid process involving two distillation columns (D-PV-D) is shown in Figure 4, and the stream conditions at key plant locations are given in Table 3. The first distillation column was similar to the previous hybrid case, where the top stream composition remained at 85 wt.% IPA and 15 wt.% water. In this process, the pervaporation module was used to break the azeotrope and dehydrate the IPA stream up to a 5 wt.% water concentration. The retentate was then fed to the second distillation column, where the target purity of 99.5 wt.% was achieved. Based on the average flux obtained, the membrane area was estimated, and the pervaporation module was sized accordingly. The downstream process equipment, such as the vacuum pump, condenser, and chiller, is also the same as in the D-PV case. The number of stages, feed stage, and reflux ratios of the columns involved are given in Table S9.

**Figure 5:**
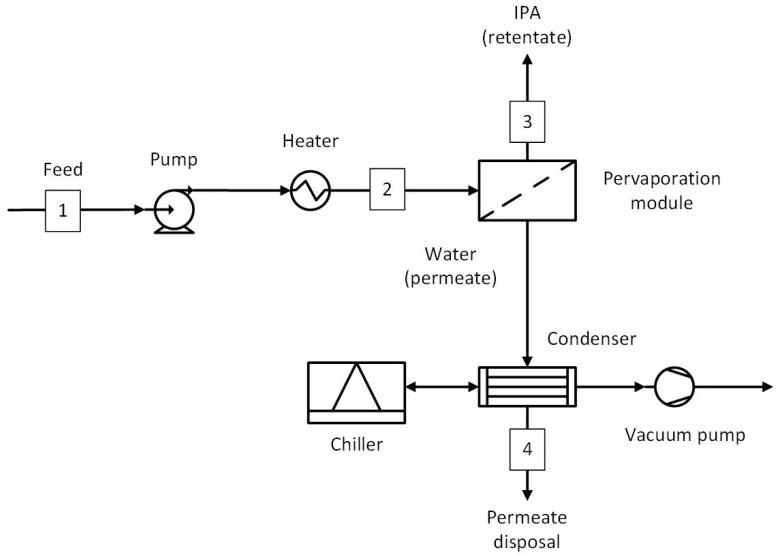
Standalone pervaporation (PV) process. Numbered streams (1–4) represent key process flows including feed, product, and recycle streams.

The standalone pervaporation system (PV) is shown in Figure 5, and the stream data at key locations are given in Table 4. The feed at the initial concentration (50 wt.% IPA and 50 wt.% water) was directly fed to the pervaporation module after raising the pressure and temperature to 6 bar and 130 °C, respectively. The permeate was condensed in the same way as the hybrid processes. However, due to the large permeate flow rate, the equipment size is expected to be relatively larger.

### 2.3. Economic Assessment Methodology

The economic assessment methodology is presented in this section, describing the calculation of capital and operating costs. The capital costs constitute equipment purchase, installation, offsite, design and engineering, and contingency costs. The operating costs comprise costs such as insurance, maintenance, and labor, which are generally fixed, and costs such as utilities, chemicals, membrane replacement, and waste disposal, which are variable and depend on the market conditions.

#### 2.3.1. Capital Costs

The capital costs of all equipment were estimated using the methodology from Sinnott and Towler [24], and using the assumptions given in Table 5. In this methodology, the equipment cost (EC) was multiplied by the appropriate installation factor to obtain the total installed cost (TIC), as shown in Table 6. The equipment costs were obtained from the Aspen process economic analyzer for all equipment except for the pervaporation module and chiller, which were obtained from respective vendors. A vacuum pump of appropriate size was modeled in Aspen Plus and used as a reference cost, along with the capital cost scaling Equation (5) to arrive at a cost specific to a particular case. The scaling exponents for all the equipment used are given in Table 6. The capital cost scaling was also used in the sensitivity analysis presented in Section 3.4. A linear membrane cost scaling assumption was applied over the studied 4–24 m^2^ range, consistent with the modular nature of commercial HybSi^®^ systems and the literature (e.g., Van Hoof et al.). Within this range, membrane prices are typically quoted per m^2^, reflecting quasi-linear behavior. A sensitivity analysis (±60% membrane cost) (see Section 3.4) showed minimal impact on LCOS trends, confirming the robustness of this assumption. The price of the pervaporation module (membrane and pressure housing) used in this study cannot be disclosed explicitly due to confidentiality agreements. Additional multipliers were added for offsite, design and engineering, and contingency costs to calculate the total cost of a greenfield plant (TPC). The offsite costs (OSs), also called outside battery limit (OSBL) costs, refer to the additional investment made in the infrastructure to accommodate a new plant or an increase in the capacity of an existing plant. These investments include electricity substations, standby generators, boilers, cooling towers, water demineralization, wastewater treatment, emergency services, firefighting equipment, landscaping, etc. In this study, off-site costs are assumed to be 30% of the TPC [24]. The design and engineering (D&E) costs refer to the cost of design and other engineering services required to carry out the project and include detailed design engineering of process equipment, procurement, construction supervision, administrative charges, etc. For the current assessment, D&E costs are assumed to be 30% of combined TIC and OS costs [24]. The contingency charges are the additional costs included in the project budget to compensate for the variations from the cost estimate, such as variations in project scope, prices, currency fluctuations, etc. A minimum contingency of 10% of combined TIC and OS costs was assumed, considering the high technology readiness of the processes under investigation [24]. Once the TPC was calculated, the annualized capital cost was estimated using the weighted average cost of capital (WACC) of 4.8% and the economic lifetime of individual equipment given in Table 6.

(5) $$Cost\;of\;equipment\;A=\left(Cost\;of\;equipment\;B\right)\times {\left(\frac{Capacity\;A}{Capacity\;B}\right)}^{Exponent}$$

#### 2.3.2. Operating Costs

Both fixed and variable operating costs were considered in the economic assessment, as shown in Table 7. In the fixed costs, insurance and maintenance were assumed to be 1.5% [32] and 2.5% [11] of the total cost of a greenfield plant (TPC), respectively. For the labor costs, 2 persons were assumed to be required to monitor and operate the plant for all cases. It is the sum of the number of persons required to operate each equipment in the plant, as listed in Table 6, rounded up. The average salary per person was assumed to be € 60,000/yr, typical of experienced plant operators in Belgium [33].

The variable operating costs assumptions are summarized in Table 7. Membranes are assumed to have a lifespan of 5 years, after which they need to be replaced. The module is assumed to have a longer lifespan, equal to the plant’s lifetime. Therefore, only the membrane cost is considered during replacement, in addition to the installation costs. The cost recovery from recycling the membranes after 5 years, either for reuse or selling, was not considered. Significant cost reductions can be achieved when repurposing the membranes for reuse. The electricity and natural gas prices follow the current energy prices in Belgium. Using the natural gas price, the medium pressure (8.75 bar and 175 °C) steam price was calculated using an online application [34]. The cooling water with a cost of € 0.32/m^3^ was used for the condensers of the distillation column with inlet and outlet temperatures of 15 °C and 25 °C. The water stream from the benchmark and the permeate stream from pervaporation processes consist of organic contaminants, making them unsuitable for regular wastewater treatment. Hence, an appropriate treatment is required before releasing these streams into public drains. The waste disposal costs, shown in Table 7, were estimated using the costs for purification and disposal [35,36].

**Table 7:** Operation and maintenance cost assumptions.

Item	Value
Operating hours	8000 h/yr
Insurance	1.5% of TPC [32]
Maintenance	2.5% of TPC [11]
Operating labor	2 persons [31]
Labor salary	60,000 €/yr/person [33]
Membrane lifetime	5 years ^a^
Electricity	€ 60/MWh [37]
Natural gas	€ 34/MWh [38]
MP steam	€ 35/t [34]
Cooling water	€ 0.32/m^3^ [32]
Waste disposal cost	€ 3.2/m^3^ [35,36]
Benzene	€ 1135/t [39]
Isopropanol	€ 1730/t [40]

^a^ Membrane lifetime was obtained from Pervatech.

### 2.4. Environmental Analysis

A simple environmental analysis was performed, estimating the annual CO_2_ emissions resulting from the use of utilities and chemicals. The CO_2_ emission intensities of electricity, steam, cooling water, and benzene used are given in Table 8. In distillation columns, the steam was used in the reboiler, and the cooling water was used in the condenser. The steam was assumed to be produced in a gas-fired boiler. The cooling water was considered to be recycled, resulting in negligible emissions. The electricity was used by feed pumps, chiller, and vacuum pump, and was assumed to have an average CO_2_ emission intensity for the Belgian electricity mix in 2023 [41]. Benzene was used as an entrainer only in azeotropic distillation, and CO_2_ emissions were only accounted for by the make-up amount. The total annual CO_2_ emissions are the sum of the quantity of utilities/chemicals and their corresponding emission intensities.

### 2.5. Performance Metrics

Several performance metrics were defined to quantify the technical, economic, and environmental performances. The recovery efficiency (*η_rec_*) and energy intensity (*ε*) shown in Equations (6) and (7) were used to quantify and compare the technical performance of the benchmark and pervaporation processes. The recovery efficiency was calculated as the ratio of the mass flow rate of the IPA outlet stream (*ṁ_IPA_outlet_*) and IPA feed stream (*ṁ_IPA_feed_*) in t/yr, whereas the energy intensity indicates the amount of energy required per unit tonne of IPA recovered. The total energy is the sum of electricity (${\dot{W}}_{el}$) and enthalpy of steam (ṁ_st_*Δh) in MWh/yr.

(6) $$\eta_{rec}=\frac{{\dot{m}}_{IPA\_outlet}\;}{{\dot{m}}_{IPA\_feed}}$$

(7) $$\varepsilon =\frac{{\dot{W}}_{el}+{\dot{m}}_{st}*\Delta h}{{\dot{m}}_{IPA\_outlet}}$$

For comparing and quantifying the economic performance, the levelized cost of separation (LCOS) in €/t IPA was used, which was calculated using Equation (8). Here, *C_capex_* and *C_opex_* represent the annualized capital (Equation (S2) in Supplementary Material) and operating costs, as outlined in previous sections in €/yr. The emission intensity (*E*) was used to quantify the plant performance in terms of CO_2_ emissions, and was calculated using Equation (9), where the numerator refers to the annual CO_2_ emissions (t-CO_2_/yr) as described in Section 2.4, and the denominator is the amount of IPA recovered annually.

(8) $$LCOS=\frac{C_{capex}+C_{opex}}{{\dot{m}}_{IPA\_outlet}}$$

(9) $$E=\frac{{\dot{W}}_{el}*E_{el}+{\dot{m}}_{st}*E_{st}+{\dot{m}}_{CW}*E_{CW}+{\dot{m}}_{B}*E_{B}}{{\dot{m}}_{IPA\_outlet}}$$

**Figure 6:**
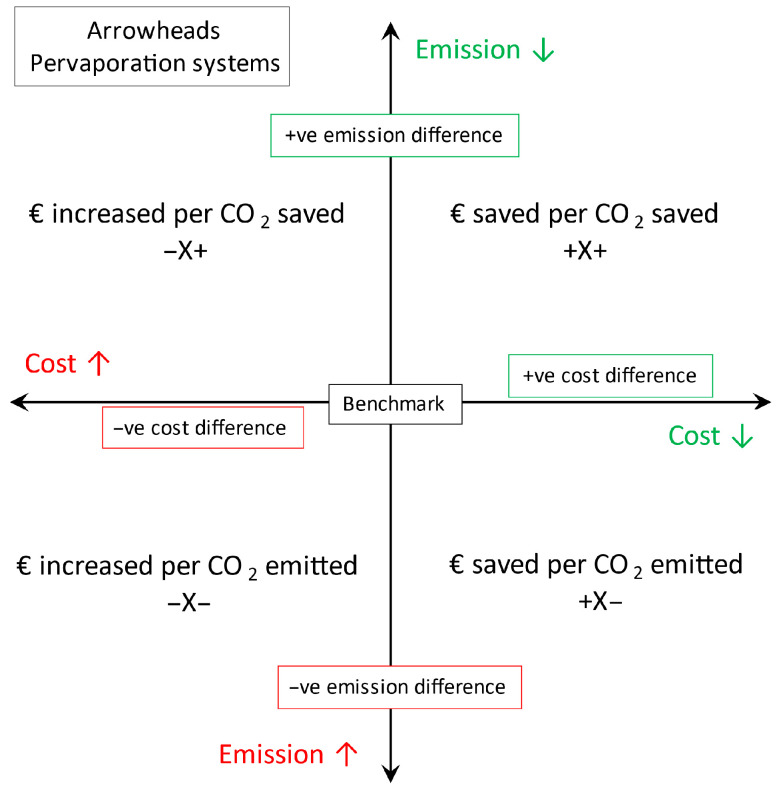
Visual interpretation of the *COPCO* index.

The pervaporation processes may be either cheaper or more expensive and may reduce or emit more CO_2_ than the benchmark. When comparing these processes, it is important to correctly express the nature of pervaporation processes in relation to the benchmark. Thus, a combined index, *COPCO*, is proposed and calculated using Equation (10) and has units of €/t-CO_2_eq. *COPCO* stands for the cost savings/expenditure of the pervaporation systems per unit tonne of CO_2_ saved/emitted. The subscript *ben* stands for benchmark process, and *per* stands for different pervaporation processes investigated in this study. This combined index is particularly useful if a company’s goal is to reduce a certain amount of CO_2_ emissions and wants to know which processes are more economical or expensive per tonne of CO_2_ saved or emitted. A visual interpretation of the four scenarios is given in Figure 6, where the resulting number (X) can be denoted in the format (−X− or −X− or −X+ or +X+). More explanation of the *COPCO* index is given in Table S4 of the Supplementary Material. Ideally, the first scenario is always desired, which is the case with the current IPA solvent under study (see Section 3.5). This may be different for different solvent systems, which will be investigated in a future study.

(10) $$COPCO=\frac{{LCOS}_{ben}-{LCOS}_{per}}{E_{ben}-E_{per}}$$

## 3. Results

Results are presented for the experimental, technical, economic, and environmental performances of the pervaporation systems. For the experimental results, the average water flux obtained at different vacuum pressures and inlet water concentrations for different pervaporation processes is discussed. In the technical analysis, the performance based on recovery efficiency, energy intensity, steam, and cooling water consumption is presented. Subsequently, the results of the economic and environmental analysis are presented in terms of LCOS, emission intensity, and *COPCO* index. Lastly, a sensitivity analysis is also presented for the parameters involving high uncertainty, such as feed flow rate, vacuum pressure, feed water content (FWC) to pervaporation, average water flux, membrane life, prices of membrane, steam, and cooling water.

### 3.1. Experimental Pervaporation Results

**Figure 7:**
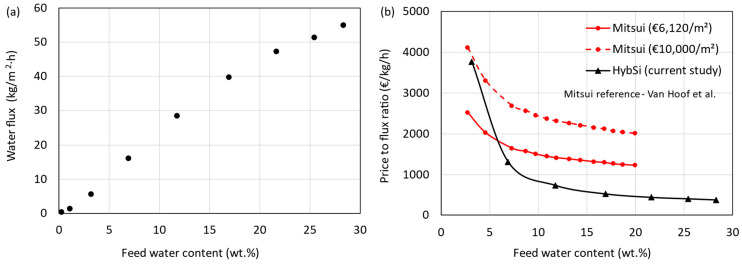
(**a**) Average flux as a function of feed water concentration. (**b**) Comparison of membrane price-to-flux ratio as a function of feed water concentration [11].

The total water flux obtained as a function of feed water content at a feed temperature of 130 °C is shown in Figure 7a. The HybSi^®^ AR membranes showed high selectivity and high flux, demonstrating their exceptional suitability for IPA dehydration. The silica network of HybSi^®^ membranes facilitates water transport through preferential sorption and diffusion, driven by molecular size and polarity differences. Operating at 130 °C increases water mobility and vapor pressure, enhancing flux while maintaining selectivity. As noted in Section 2.1, the driving force in pervaporation is the partial pressure gradient of water across the membrane. Elevated temperatures raise the feed-side partial pressure, thereby intensifying the driving force and resulting in higher permeation rates. The fluxes of Mitsui membranes reported by Van Hoof et al. [11] are significantly lower than the current HybSi^®^ AR membrane (e.g., 5 times lower at 10 wt.% water). Flux increases nonlinearly with feed water content due to Raoult’s law effects and enhanced sorption-driven transport. Similarly, the price of the Mitsui membrane (€ 3400/m^2^) reported was also lower than the HybSi^®^ AR membranes. The current price of Mitsui membranes was not available. Therefore, the prices were updated to the current year using the chemical engineering plant cost index (CEPCI) (2004-444.2 and 2024-800.3), resulting in a price of € 6120/m^2^. However, using CEPCI may not be the right index to use, considering the increase in material, manufacturing, and labor costs in the past decades. A comparison between the Mitsui and HybSi^®^ AR membranes can be made by considering the price-to-flux ratio (€/kg/h) as a function of feed water content, as shown in Figure 7b. The flux values of Mitsui membranes at 90 °C were extracted from Van Hoof et al. [11]. These fluxes were used with the updated price (€ 6120/m^2^) to calculate the price-to-flux ratios represented by the red continuous line in Figure 7b. The black continuous line represents the price-to-flux ratio for the HybSi^®^ AR membrane. The results show that the HybSi^®^ AR membrane performs better for most feed water contents. At a feed water content below about 6 wt.%, the Mitsui membranes showed better performance. However, as mentioned earlier, the actual membrane price could be higher than the estimation based on the Mitsui membrane price reported by Van Hoof et al. [11]. Considering a price of € 10,000/m^2^, the performance of HybSi^®^ AR membranes is better at all feed water contents. Table S13 compares our system to prior work (Van Hoof et al. [11] and Harvianto et al. [12]), showing superior flux and competitive LCOS. This comparison highlights the competitive performance of our HybSi^®^-based system, particularly at low water concentrations, and supports the feasibility of the proposed hybrid configurations.

Table 9 shows the average flux, permeate water concentration, and membrane surface area requirement at various vacuum pressures, feed water contents, and product water contents. The permeance and selectivity values were also calculated at representative operating conditions and are reported in Table S15 of the Supplementary Material. Permeance ($Q_{H_{2}O}$) was defined as the molar flux divided by the partial pressure difference across the membrane, while selectivity (or separation factor) was defined as the ratio of product water-to-IPA concentrations in the permeate and feed, respectively. These formulations are consistent with established definitions in pervaporation and membrane science [1,43,44]. The experiments were performed until 30 wt.% of water concentration and extrapolated further until 50 wt.% water concentration using the VLE relation data. The organic flux was assumed to be 0.1 kg/h∙m^2^ for all cases due to difficulties in determination. It can be seen that the average flux values are greatly dependent on the aforementioned parameters. When the vacuum pressure increases, the average flux decreases, irrespective of the feed water concentration. A higher flux at lower permeate pressures (20 mbar) is due to a higher driving force, while at higher permeate pressures (50 mbar), the driving force is reduced, leading to a lower flux [45]. Furthermore, the operating vacuum pressures also affect the evaporation rate of the permeate. At lower pressures, the evaporation rate of water is enhanced, leading to higher fluxes [46]. The feed water content (water in) and retentate water content (water out) also have a significant influence on the average flux values, as shown in Figure 7a and Table 9. This is due to the difference in driving force resulting from the concentration gradient across the membrane. For the hybrid D-PV and D-PV-D processes, water in (wt.%) represents the top stream water content of column 1. For example, when the feed water content increases from 15 wt.% (D-PV) to 50 wt.% (PV), the average flux values increase by almost 70% at both vacuum pressures. This increase is not reflected in the required membrane area, as it also depends on the permeate flow rate. The permeate quality (or selectivity) is also listed in Table 9 and seems to be dependent on the average flux. The higher the flux, the better the permeate quality, meaning that the loss of organic solvent is minimal, resulting in high IPA recovery efficiencies. The high permeate quality indicates the high selectivity of the HybSi^®^ AR membranes toward water.

### 3.2. Technical Performance

The technical performance of the azeotropic distillation process and the three pervaporation-based processes is presented in Table 10. The membrane area required was calculated based on the average water flux and the permeate flow rates. Depending on the size of the process, the number of membrane modules can be estimated based on the standard module size of 3 m^2^, which is readily available. However, a design for 10 m^2^ modules is also available on the market. Several modules are arranged in series to achieve the desired retentate purity. For the D-PV process, a total of 11 m^2^ area is required, resulting in three modules of 3 m^2^ area and 1 module of 2 m^2^ area. For the D-PV-D process, since the retentate purity required was restricted to 5 wt.% water, the membrane area required was just 4 m^2^, resulting in one module of 3 m^2^ area and another one of just 1 m^2^ area. For the standalone PV process, the membrane area required was 19 m^2^, resulting in 6 modules of 3 m^2^ and 1 module of 1 m^2^. The recovery efficiencies of pervaporation-based processes are on par with the benchmark process. The highest recovery efficiency was observed for the D-PV-D process, as it involved a second distillation column after the pervaporation module.

Steam is the primary energy source that is used in reboilers of distillation columns, feed heating to operating temperature, and interstage heating in pervaporation processes. A boiler may be used on-site to generate this steam; however, only the costs associated with producing this steam were considered in this study. The interstage heating was carried out after each module to reheat the retentate to 130 °C before the next pervaporation module. For simplicity, it was assumed that the heat required in the interstage heating was equivalent to the heat of vaporization of the entire permeate flow rate. In the benchmark process (D-D-D), only steam was considered as the energy consumption. The combined steam consumption shown in Table 10 is divided among the three columns in a ratio of 41:16:1. The most steam was consumed in column 1, as it has to heat the mixture to a near-azeotropic boiling point (~80 °C). In column 2, the top stream was a mixture of IPA, water, and benzene, forming a ternary azeotrope, and also had a temperature near their azeotropic point (~62 °C). The least steam was needed in column 3, which was used to recover benzene in the top stream at 10 kg/h (Figure 2 and Table 1). The D-PV process required 58% less steam than the benchmark, most of which was used in column 1 (~80%), where the top stream was brought to near azeotropic conditions (80 °C). The rest of the steam was used in feed heating (8%) and interstage heating (12%). For the D-PV-D process, column 1 used 41% of the steam while column 2 used 46% as it handles larger flow rates (see Figure 4). The feed and interstage steam requirements were 7% and 6%, respectively. In the standalone PV, about 30% of the steam was used for feed heating, while the remaining steam was used for interstage heating. If a boiler was used on-site to produce the required steam, the D-PV and PV processes would have smaller-sized boilers compared to the benchmark.

The electricity consumption was neglected for the azeotropic distillation process since all distillation columns operated at atmospheric pressure. The only electricity required was for pumping, which was not considered in the current study. The electricity required in pervaporation-based processes was mainly consumed by the chiller required for permeate condensation (>90%), followed by the vacuum pump and feed pump. For the D-PV and D-PV-D processes, the electricity consumption was similar since the permeate flow rates are the same. The electricity consumption of the standalone PV process was 884 MWh/yr, about 82% more than the hybrid processes, due to larger permeate flow rates. In this case, about 98% of the electricity was consumed by the chiller.

Cooling water was required for the condensation of the distillate in the columns and the vacuum pump. Depending on the solvent system under consideration, cooling water may also be required for condensing the permeate instead of a chiller. This may reduce the separation costs resulting from eliminating the capital and electricity costs of the chiller in certain cases. However, in the current study, using cooling water for permeate condensation was not feasible due to a low vacuum pressure of 20 mbar at which the saturation temperature was ~17 °C. Compared to the benchmark, the cooling water consumption by D-PV, D-PV-D, and PV processes was 72%, 24.6%, and 99.9% less. The heat accumulated by the cooling water is generally rejected in a cooling tower integrated with the base plant, requiring additional auxiliary equipment and land. The less cooling water is consumed, the less piping and recycling equipment are required. The PV process uses just above 1 m^3^/day of cooling water and would not require a dedicated cooling tower compared to the benchmark and the hybrid cases.

To have a proper comparison in terms of energy consumption, energy intensity was used, which combines both electricity and steam into one metric, as discussed in Section 2.5. The benchmark case consumed about 2 MWh/t-IPA, whereas the D-PV, D-PV-D, and PV cases consumed 56%, 16%, and 46% less energy, respectively. For the benchmark case, all energy comes from steam, whereas for the hybrid and PV cases, the majority of the energy comes from steam.

### 3.3. Economic Performance

**Figure 8:**
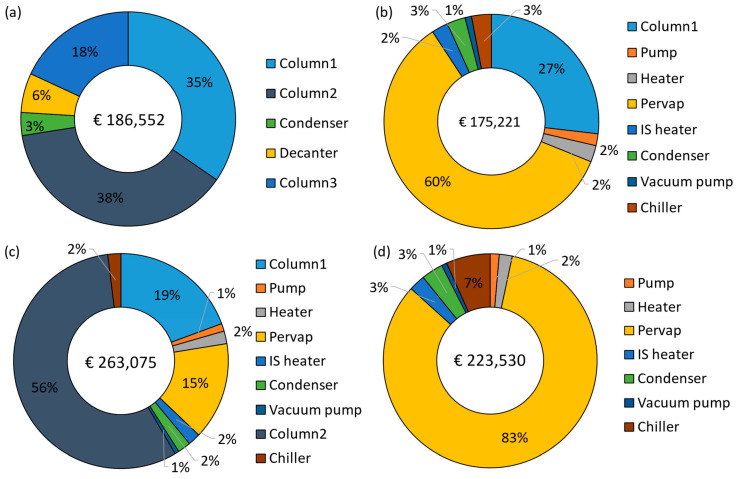
Annualized capital cost breakdown: (**a**) D-D-D, (**b**) D-PV, (**c**) D-PV-D, and (**d**) PV.

The annualized capital costs and their breakdown are shown in Figure 8, with the equipment names corresponding to Figure 2, Figure 3, Figure 4 and Figure 5. The capital costs were estimated for the feed capacity of 1000 kg/h and included all equipment related to distillation and pervaporation units. It also includes the installation, off-site, design and engineering, and contingency costs. The distillation unit includes a tower, reboiler, condenser, overhead accumulator, and reflux pump, while the pervaporation unit includes a feed pump, heater, pervaporation modules including membranes, interstage heater, condenser, vacuum pump, and a chiller. The *IS heater* in Figure 8a–c refers to the interstage heater, which is not shown in the process flow diagrams. It is considered one big heater heating the entire permeate stream instead of smaller heaters after each module. The *Pervap* in Figure 8a–c consists of only the initial membrane costs, while the replacements were included in the operating costs.

The capital cost of the benchmark was € 186,552/yr, where the majority of the cost comes from distillation columns (91%), with column 2 (azeotropic) accounting for 38%, followed by column 1 at 35% and column 3 at 18%. The capital cost of the D-PV process stands at € 175,221/yr, about 6% less than the benchmark. About 60% of the cost comes from the pervaporation module, followed by 27% from column 1. The cost considered for the HybSi^®^ AR membranes is relatively high and thus results in capital costs close to the benchmark. This is different from Van Hoof et al. [11], where comparatively cheaper Mitsui membranes were considered, which resulted in an almost 50% reduction in capital costs. The capital cost estimate for the D-PV-D process was 41% more expensive than the benchmark, while it was 33.4% more expensive than the D-PV process due to the contribution from the second column. About 56% is attributed to column 2 as it handles larger flows (874 kg/h) due to the top recycle stream (see Table 3), followed by column 1 at 19%. The pervaporation module contributes only 15% due to lower membrane area requirements. In Van Hoof et al. [11], unlike the current study, the D-PV-D process still had lower capital costs compared to the benchmark, probably due to the differences in the cost considerations. The standalone PV process has a capital cost of € 223,530/yr, which is 16.5% more than the benchmark. Compared to hybrid processes, the capital cost of the PV process is 21.6% more than the D-PV and 15% less than the D-PV-D. The capital cost is higher due to the cost of HybSi^®^ AR membranes, which account for 83% of the total capital cost, but still less than the contribution from distillation columns in the hybrid D-PV-D process. Furthermore, a big chiller was required in the PV process as it condenses a large permeate flow rate (499.4 kg/h).

Space requirements are sometimes critical in selecting or designing appropriate separation processes. The cost associated with the spatial footprint may have an additional impact on the total capital costs. However, due to limited information available on the space requirements, these costs were excluded from the assessment. The distillation columns require more space and large buildings, depending on the column diameter and height. They also require substantial space to accommodate the reboilers, condensers, piping, and supporting infrastructure such as pumps and control systems. The pervaporation systems are more compact compared to distillation columns and can be arranged in a smaller footprint. These systems require less vertical space, making them suitable for locations with height constraints. Furthermore, since they are modular, scaling and integration into existing systems is relatively easy. This is the reason why the capital cost scaling of pervaporation systems was assumed to be linear in the current assessment.

**Figure 9:**
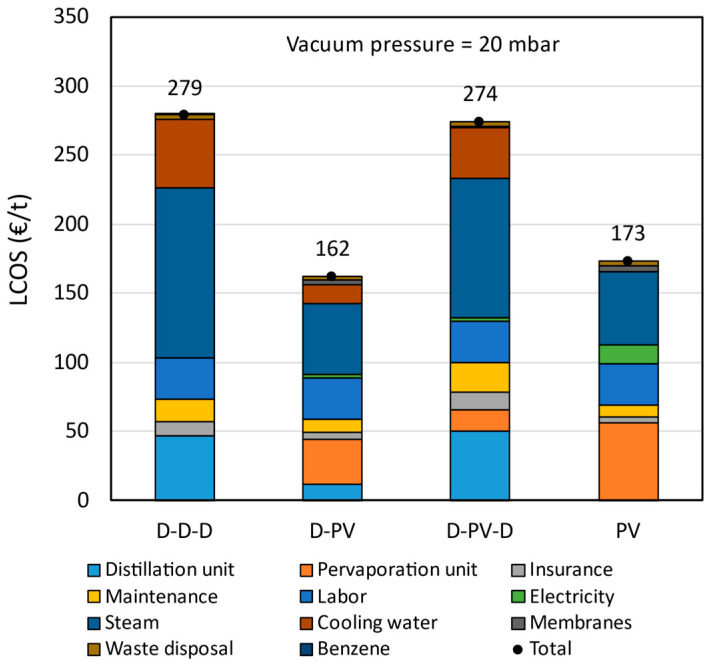
Levelized cost of separation breakdown at VP = 20 mbar and FWC = 15 wt.%.

The LCOS breakdown of the pervaporation-based processes compared to the benchmark is shown in Figure 9. The capital costs are represented by the distillation and pervaporation units, while all the other cost elements represent operating costs. The fixed operating costs (insurance and maintenance) are dependent on the capital costs, and hence, the higher the capital cost, the higher the fixed operating costs. The labor costs are the same in all cases, as it was assumed that two persons are required to operate and monitor the plants. The energy (steam and electricity) accounts for the largest share of the levelized costs. The cooling water costs are also significant in cases where more distillation columns are present, such as the benchmark, followed by D-PV-D and D-PV processes.

The LCOS calculated for the benchmark azeotropic distillation is € 279/t, which is 53.4% more than that reported by Van Hoof et al. [11]. The current energy prices, equipment costs, and additional but essential cost elements, such as insurance, labor, waste disposal, etc., contributed to such an increase in LCOS. The hybrid D-PV process has an LCOS of € 162/t, which is 41.8% less than the benchmark. This is mainly due to a reduction in steam and cooling water consumption, and partly due to a reduction in capital costs. The LCOS of the hybrid D-PV-D process is € 274/t, almost the same as the benchmark. In this process, there is a reduction in operating costs by 10% due to lower steam and cooling water consumption. However, the presence of two distillation columns and a pervaporation unit increases the capital costs by 29% which is also reflected in fixed operating costs. For the standalone PV process, the LCOS is € 106/t (or 37.8%) lower than the benchmark. It only consists of the pervaporation unit, and due to large membrane area requirements, the capital costs are 17% higher than the benchmark. However, there is a significant reduction in steam and cooling water consumption, resulting in lower LCOS. Based on the LCOS, the hybrid D-PV and standalone PV are better options for a greenfield plant and better alternatives for an azeotropic distillation plant.

If the distillation columns are already available, then the D-PV process would be better suited since the existing distillation column can be reused after modifications, and the pervaporation unit can be retrofitted without any difficulty. In this case, the LCOS would be € 144/t (after removing column capital cost) as opposed to € 162/t when all the equipment is accounted for. On the contrary, if a new installation is being planned, then the standalone PV is the better option since it requires less space and is well-suited to be a greenfield plant. The D-PV-D process is useful when retrofitting an existing distillation plant (brownfield) where the columns can be repurposed to the required specifications. Only a smaller pervaporation unit is required as a replacement for the azeotropic column (column 2) of the benchmark. Here, the LCOS would be € 196/t when the capital cost of two columns is excluded.

Significance analysis was conducted by comparing key performance metrics across configurations, with maximum percentage differences calculated to highlight meaningful gaps. As shown in Table S14, LCOS varied by up to 69% and CO_2_ emissions by 489%, indicating substantial differences in economic and environmental performance.

### 3.4. Sensitivity Analysis

**Figure 10:**
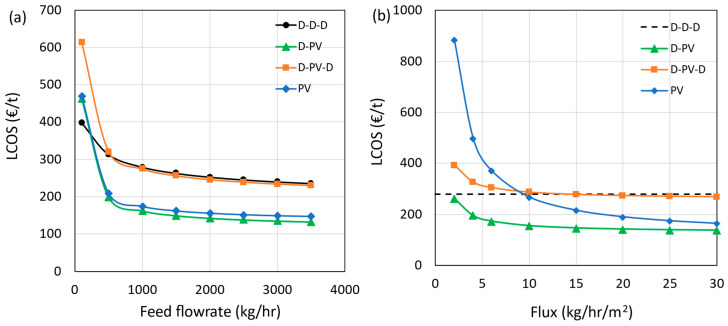
Impact of feed flow rate (**a**) and flux (**b**) on LCOS.

The sensitivity of the LCOS to two key technical and four key economic parameters is presented in this section. The results are presented only for greenfield plants, and no retrofitting or use of existing distillation columns was considered. The technical parameters selected are the feed flow rate (kg/h), representing the plant capacity to separate IPA and water, and the average water flux (kg/h/m^2^), representing the removal of water as a permeate per unit time and membrane area. The economic parameters selected are the membrane module price (% increase or decrease), membrane life (yr), steam (€/t), and cooling water prices (€/m^3^). The feed flow rate was varied from 100 to 3500 kg/h, with 1000 kg/h as the base value, and the results are presented in Figure 10a. Generally, the higher the feed flow rate, the lower the LCOS due to economies of scale. However, beyond 3000 kg/h, the effect of economies of scale diminishes for all cases. It is important to note that the pervaporation modules are assumed as modular and were scaled linearly. The hybrid D-PV and standalone PV processes perform better than the benchmark at all feed flow rates, indicating the cost advantage of having larger pervaporation units. On the contrary, the D-PV-D process with a smaller pervaporation unit has a similar LCOS as the benchmark. However, this trend changes at lower flow rates, as shown in the figure. At a flow rate of 100 kg/h, the benchmark case performs better than the hybrid D-PV and PV processes, followed by the D-PV-D. However, at such flow rates, operating a distillation column may pose challenges such as heat losses due to a high surface area to volume ratio, control and stability issues, etc. Therefore, a standalone PV is the best option at low flow rates. Scaling up from a lab-scale membrane to industrial-scale modules introduces several technical challenges. The key issues include maintaining uniform flux and membrane integrity over larger areas, ensuring equal flow distribution and manageable pressure drops across modules, and implementing effective interstage heat recovery. Long-term membrane stability, fouling control, and sustaining low vacuum over large permeate volumes are also critical. While HybSi^®^ AR membranes benefit from modular scalability, industrial packaging formats (e.g., spiral-wound, multi-channel ceramics) may influence transport performance. These considerations are essential for future pilot-scale implementation.

Figure 10b shows the sensitivity results when the average permeate flux was varied from 2 to 30 kg/h∙m^2^ with different base values for different processes (see Table 9). As no membranes are involved in azeotropic distillation, the LCOS is shown as a dotted line in the figure. The flux values were used to calculate the required membrane area, which is reflected in the capital and operating costs. The D-PV process has the cost advantage at all considered flux values, whereas the D-PV-D process shows no benefit even at higher flux values. The processes with a higher membrane area requirement are more sensitive to flux values, which is evident from the standalone PV results. The LCOS crosses the benchmark limit at a flux below 10 kg/h∙m^2^, indicating the advantage of membranes with a high permeate flux. The fluxes reported by Van Hoof et al., especially for Mitsui membranes, were below 5 kg/h∙m^2^. Using this flux value and an assumed current price of €10,000/m^2^, only the D-PV case emerged as the better option with an LCOS of € 155/t (D-PV-D = € 287/t and PV = € 263/t). Furthermore, the spatial footprint is also based on the required membrane area, which is, in turn, dependent on membrane flux. Higher fluxes are desired to have a compact pervaporation unit, resulting in a reduction in LCOS.

**Figure 11:**
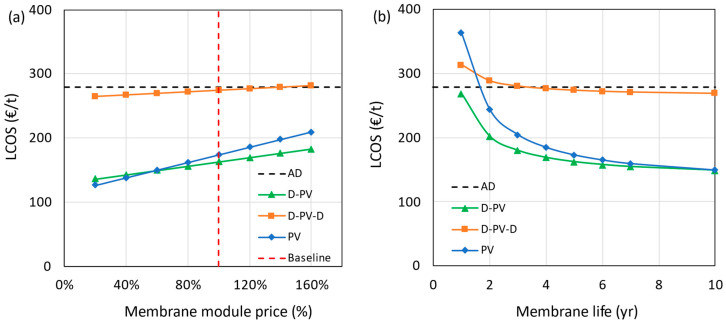
Impact of membrane module price (**a**) and lifetime (**b**) on LCOS.

The impact of the membrane module price on the LCOS is shown in Figure 11a, in which the membrane module price is varied from 20% to 160% of the base value. The effect of price is more pronounced in processes with larger membrane area requirements (D-PV and PV). The results show that the standalone PV process becomes more expensive than the D-PV process at a 60% or more reduction in membrane price. At this point, the plant could be modified to a D-PV process by retrofitting it with a distillation column. For the D-PV-D process, the LCOS was reduced by just 5% even when the membrane module price was reduced by 80%.

The membrane lifetime was varied from 1 to 10 years with a base value of 5 years, and the effect on LCOS is shown in Figure 11b. The HybSi^®^ AR membranes were reported to perform well even after 3 years when tested at a temperature of 150 °C [45]. Considering the lower operating temperature of 130 °C in the current study and with proper maintenance, the membrane lifetime could extend beyond 5 years, which has been corroborated by the experts at Pervatech. The hybrid D-PV process performs better even at a very low membrane lifetime of 1 year, while the standalone PV process needs membranes with a lifetime longer than 1.5 years for it to be competitive with the benchmark. At a hypothetical membrane lifetime of 10 years, both D-PV and PV processes converged to a common LCOS. Having a longer membrane lifetime does not have a significant impact on the LCOS of the D-PV-D process due to the lower contribution from the pervaporation membrane costs.

**Figure 12:**
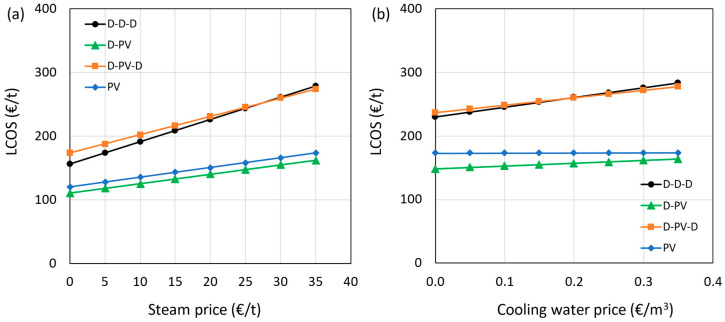
Impact of steam (**a**) and cooling water (**b**) prices on LCOS.

The effect of steam and cooling water prices on the LCOS is shown in Figure 12a,b, respectively. The steam was assumed to be produced by a gas-fired boiler and is dependent on the natural gas (NG) market price, which is highly uncertain. The distillation-heavy configurations (e.g., D–PV, D–PV–D) are more affected by natural gas price fluctuations due to higher reboiler duty. In contrast, membrane-dominant systems like PV only exhibit greater resilience due to their lower heat requirement. Therefore, adopting processes that run more on electricity and less on thermal energy is desired, which is evident from the results shown in the figure. The hybrid D-PV and standalone PV processes are less sensitive to steam and cooling water prices due to their lower consumption. The cases with two or more distillation columns, such as the benchmark D-D-D and the hybrid D-PV-D processes, consume more steam and cooling water, and are thus more sensitive to their prices. Even considering zero cost for steam, such as in the case of utilizing waste heat, the hybrid D-PV and standalone PV show lower LCOS than the benchmark.

### 3.5. Environmental Performance

The annual CO_2_ emissions, emission intensity, and *COPCO* index for all cases are shown in Table 11. The benchmark D-D-D has the highest CO_2_ emissions, followed by hybrid D-PV-D (22.5% less) and D-PV (67.5% less) processes. The lowest CO_2_ emissions were from standalone PV, which were 86% less than the benchmark due to a relatively lower energy consumption. The benchmark process emits 6.8 t-CO_2_/t-IPA, whereas the standalone PV has the lowest emission intensity of 0.9 t-CO_2_/t-IPA. If retrofitting of existing distillation columns is considered, then the hybrid D-PV process is the best option compared to the hybrid D-PV-D. The optimal choice among the three pervaporation-based processes becomes clearer when the environmental performance is combined with the cost reductions using the *COPCO* index. According to the convention discussed in Section 2.5, all three processes result in savings in both expenses and CO_2_ emissions. The processes D-PV, D-PV-D, and PV save € 25, € 3, and € 18, respectively, for every tonne of CO_2_ saved. The hybrid D-PV process is the best option both as a greenfield plant and as a retrofit.

**Figure 13:**
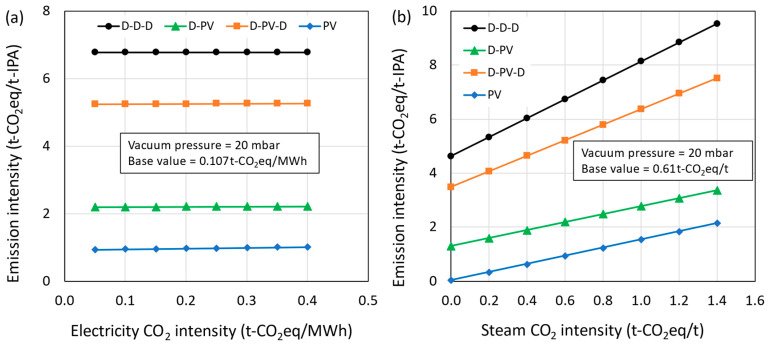
CO_2_ emission intensity as a function of (**a**) electricity and (**b**) steam CO_2_ intensity.

Considering the uncertainty in the CO_2_ emission intensities of electricity and steam based on location and energy mix, a sensitivity analysis was performed, and their effect on CO_2_ emissions is presented in Figure 13a,b, respectively. The results show that the effect of CO_2_ intensity of electricity is negligible on the overall CO_2_ emissions, as the contribution from electricity is very small (D-D-D = 0%; D-PV = 0.2%; D-PV-D = 0.1%; PV = 2.5%). The majority of energy requirement was fulfilled by steam, having a significant effect on the overall CO_2_ emissions and representing 32%, 41%, 33%, and 97% share of total CO_2_ emissions in D-D-D, D-PV, D-PV-D, and PV processes, respectively. The rest of the emissions were from cooling water usage. Different fuels such as natural gas, coal, biogas, etc., can be used to generate steam. More often, natural gas is used as a fuel since it is cleaner than other fossil fuels. The natural gas composition, plant location, and boiler efficiency may differ and affect the CO_2_ emission intensity significantly. Also, when waste heat is recovered and utilized for steam generation, the emission intensity could be different.

### 3.6. Impact of Vacuum Pressure and Feed Water Content

Table 12 presents how different operating conditions and processes affect the performance of the pervaporation systems. The effect of higher vacuum pressure (50 mbar) was investigated for all pervaporation-based systems, while the feed water content (15 wt.% to 30 wt.%) was tested only for the hybrid D-PV process. Here, the feed water content is associated with the pervaporation system, which is the same as the top stream water content of column 1 in the hybrid D-PV and D-PV-D processes. The permeate saturation temperature in the condenser was 32 °C at 50 mbar, indicating the feasibility of using cooling water instead of a chiller. The results obtained at 50 mbar can be compared with the results obtained at 20 mbar using Table 10, Figure 9, and Table 11. At higher vacuum pressures, the required membrane area increases due to a decrease in average flux. This, in turn, is due to a decrease in the driving force, which is the difference in partial vapor pressures across the membrane. A larger membrane area would increase the capital and operating cost of the pervaporation unit. Similarly, the permeate quality (selectivity) also decreases (see Table 9), which is also reflected in the recovery efficiencies. The electricity consumption was significantly lower due to the use of cooling water instead of a chiller, while the steam consumption remained almost the same at 50 mbar (see Table S10). There is also a slight reduction in product output due to relatively more IPA losses in the permeate. However, the significant reductions in electricity consumption due to the omission of a chiller improve the energy intensities at 50 mbar. Lowering permeate pressure to 20 mbar enhances selectivity and flux but increases cooling energy. Operating at 50 mbar allows for passive condensation but reduces separation efficiency.

When LCOS is compared, there is an increase of 8.4%, 0.5%, and 8.7% for D-PV, D-PV-D, and PV processes, respectively. The breakdown of LCOS is shown in Figure S1 of the Supplementary Material. The increase was primarily due to the high cost of the pervaporation unit, caused by the need for a larger membrane area due to lower flux at 50 mbar. The reduction in electricity consumption did not positively impact the LCOS. The emission intensities were higher than at 20 mbar pressure because of increased cooling water consumption, which is the main driver of emissions. Another advantage of having 50 mbar vacuum pressure is the reduction in spatial footprint associated with a chiller, which was not considered in the current assessment. The *COPCO* index shows that all the processes still save money per tonne of CO_2_ saved, where D-PV emerged as the best option again.

The impact of varying the feed water content in the D-PV process was investigated, and the results obtained are shown in Table 12. An increase in feed water content results in an increase in average water flux (see Table 9). Due to an increase in the flow rate to the pervaporation system, several changes were observed. The required membrane area increased slightly, and electricity consumption by the feed pump, chiller, and vacuum pump also increased. Additionally, steam consumption in the feed and interstage heating increased, leading to higher energy intensity. The capital cost of the distillation unit went up due to the need for a larger column to obtain more water at the top. The capital cost of the pervaporation unit also increased. Consequently, both capital and operating costs increased, resulting in a higher LCOS. Increased steam and cooling water consumption led to higher emission intensity, ultimately resulting in a decrease in the *COPCO* index. It is evident from the results that the feed water content should be close to the azeotropic point for a process to be the best option economically and environmentally.

## 4. Conclusions

This study presents the first combined techno-economic and environmental analysis of IPA dehydration using HybSi^®^ membranes across three configurations. A new cost-efficiency metric, *COPCO*, is introduced, alongside updated modeling under 2024 market conditions. These processes showed a substantial reduction in the levelized cost of separation and CO_2_ emissions when compared to traditional azeotropic distillation. For the pervaporation processes, HybSi^®^ AR membranes were used, which showed exceptional solvent recovery and selectivity. The hybrid distillation-pervaporation (D-PV) process with just one distillation column achieved the highest cost reduction of 42% along with a CO_2_ emission reduction of 68%. This was followed by the standalone pervaporation (PV), which showed a cost reduction of 38% and a CO_2_ emission reduction of 86%. The hybrid distillation-pervaporation-distillation (D-PV-D) process, combining pervaporation with two distillation columns, showed minor savings in cost (<2%) while the savings in CO_2_ emissions were 22%. The *COPCO* analysis revealed that the D–PV configuration offers the highest cost-efficiency among the evaluated systems, outperforming PV-only and D-PV-D configurations by effectively balancing membrane cost, energy demand, and recovery performance. The hybrid D-PV process emerged as the best alternative to the azeotropic distillation process, offering several technical, economic, and environmental advantages. The cost savings are substantial when compared to purchasing virgin IPA at the market price of € 1730 per tonne. The sensitivity analysis showed that lowering permeate pressure to 20 mbar enhances selectivity and flux but increases cooling energy. Operating at 50 mbar allows for passive condensation but reduces separation efficiency. The results of this study are expected to address the need to replace, debottleneck, or intensify traditional distillation-based processes for solvent recovery within the chemical, pharmaceutical, and related industries. Preliminary data suggest that HybSi^®^ membranes also hold promise for other solvent mixtures such as tetrahydrofuran–water, acetonitrile–water, etc., with ongoing studies focused on optimizing conditions. Results will be reported in future work. Expanding the use of pervaporation in these markets will benefit both these industries and society by reducing energy consumption and, consequently, lowering the industry’s carbon footprint.

## Figures and Tables

**Table 1:** Stream conditions at selected locations in the azeotropic distillation process (Figure 2).

Stream	Temperature	Mass Flow Rate	IPA	H_2_O	C_6_H_6_
	°C	kg/ h	wt.%	wt.%	wt.%
1	20	1000	50.0%	50.0%	0.0%
2	80	716	86.5%	13.5%	0.0%
3	99	499	0.2%	99.8%	0.0%
4	62	1365	18.6%	11.4%	70.1%
5	82	501	99.5%	0.5%	0.0%
6	60	10	18.6%	7.6%	73.8%
7	81	215	56.2%	43.8%	0.0%
8	20	1150	11.5%	5.4%	83.2%
9	20	0.015	0.0%	0.0%	100.0%

**Table 2:** Stream conditions at selected locations in the D-PV process (Figure 3).

Stream	Temperature	Mass Flow Rate	IPA	H_2_O
	°C	kg/h	wt.%	wt.%
1	20	1000	50.0%	50.0%
2	80	587	85.0%	15.0%
3	99	413	0.2%	99.8%
4	130	587	85.0%	15.0%
5	130	498	99.5%	0.5%
6	17	87	1.2%	98.8%

**Table 3:** Stream conditions at selected locations in the D-PV-D process (Figure 4).

Stream	Temperature	Mass Flow Rate	IPA	H_2_O
	°C	kg/h	wt.%	wt.%
1	20	1000	50.0%	50.0%
2	80	587	85.0%	15.0%
3	99	413	0.2%	99.8%
4	130	960	86.5%	13.5%
5	130	874	95.0%	5.0%
6	17	86	0.5%	99.5%
7	80	372	88.9%	11.1%
8	82	501	99.5%	0.5%

**Table 4:** Stream conditions at selected locations in the PV process (Figure 5).

Stream	Temperature	Mass Flow Rate	IPA	H_2_O
	°C	kg/h	wt.%	wt.%
1	20	1000	50.0%	50.0%
2	130	1000	50.0%	50.0%
3	130	501	99.5%	0.5%
4	17	499	0.4%	99.6%

**Table 5:** Capital costs assumptions.

Item	Value
Base year	2024
Equipment cost (EC)	Aspen
Total installed cost (TIC)	EC x Installation factor
Offsites (OS)	30% of TIC [24]
Design and engineering (D&E)	30% of TIC + OS [24]
Contingency (X)	10% of TIC + OS [24]
Total plant cost (TPC)	TIC (1 + OS) (1 + D&E + X) [24]
Chiller reference cost	€17,655 [25]
Chiller reference capacity	42 kW [25]
Vacuum pump reference cost	€60,600 ^a^
Vacuum pump reference capacity	18.5 kg/h∙kPa ^a^
Plant lifetime	25 yr
WACC	4.8% ^b^

^a^ Vacuum pump modeled for the given size available in Aspen. ^b^ Discount rate = 9%, equity = 20%, debt = 80%, tax rate = 25%, interest rate = 5%. See Equation (S1).

**Table 6:** Economic assumptions of the installation factor, scaling exponent, lifetime, and labor requirement.

Component	Installation Factor	Scaling Exponent	Lifetime	Labor
	-	-	(yr)	(Per Shift)
Column	4 ^a^	0.78 ^b^	25 ^f^	0.5 ^g^
Pump	4 ^a^	0.6 ^b^	25 ^f^	0.1 ^g^
Heater	3.5 ^a^	0.68 ^b^	25 ^f^	0.1 ^g^
Decanter	4 ^a^	0.57 [26]	25 ^f^	0.2 ^g^
Membrane	2 [11]	1 ^c^	5 ^e^	0.04 ^e^
Interstage heater	3.5 ^a^	0.68 ^b^	25 ^f^	0.1 ^g^
Condenser	3.5 ^a^	0.68 ^b^	25 ^f^	0.1 ^g^
Vacuum pump	1.7 [27]	0.75 ^b^	25 [28]	0.1 ^g^
Chiller	1.6 ^b^	0.6 ^d^	20 [29]	0.1 ^g^

^a^ [24]; ^b^ [30]; ^c^ modular; ^d^ assumption; ^e^ Pervatech; ^f^ average lifetime; ^g^ [31].

**Table 8:** CO_2_ emission intensity of utilities and chemicals.

Utility	CO_2_ Emission Intensity (t-CO_2_eq/unit)
Electricity	0.107/MWh [41]
Steam	0.61/t [42]
Cooling water	0.03/t [42]
Benzene	1.76/t [42]

**Table 9:** Permeate flux and quality.

Case	Vacuum Pressure	Water In	Water Out	Avg. Water Flux	Permeate Quality
	mbar	wt.%	wt.%	kg/m^2^∙h	wt.%
D-PV	20	15%	0.5%	8.0	98.77%
D-PV-D	20	15%	5%	21.5	99.54%
PV	20	50%	0.5%	25.84	99.61%
D-PV	50	15%	0.5%	5.43	98.19%
D-PV-D	50	15%	5%	17.67	99.44%
PV	50	50%	0.5%	20.42	99.51%
D-PV	20	20%	0.5%	10.54	99.06%
D-PV	20	25%	0.5%	13.15	99.25%
D-PV	20	30%	0.5%	15.85	99.37%

**Table 10:** Technical performance metrics and utility consumption (VP = 20 mbar; FWC = 15 wt.%).

Metric	D-D-D	D-PV	D-PV-D	PV
Membrane area (m^2^)	-	11	4	19
Recovery efficiency (%)	99.7%	99.6%	99.8%	99.6%
Steam (t/yr)	14,082	5914	11,557	6059
Electricity (MWh/yr)	0 *	164	163	884
Cooling water (m^3^/yr)	619,021	173,323	466,523	421
Energy intensity (MWh/t-IPA)	2.0	0.9	1.7	1.1

* Electricity required for pumping was negligible and neglected; VP—vacuum pressure; FWC—feed water content.

**Table 11:** Emission intensity and *COPCO* index at VP = 20 mbar and FWC = 15 wt.%.

Case	Annual Emissions	Emission Intensity	*COPCO* Index
	t-CO_2_eq/yr	t-CO_2_eq/t-IPA	€ /t-CO_2_eq
D-D-D	27,161	6.8	-
D-PV	8825	2.2	+25+
D-PV-D	21,063	5.3	+3+
PV	3803	0.9	+18+

**Table 12:** Impact of VP and FWC on technical, economic, and environmental performance metrics.

Case	D-PV	D-PV-D	PV	D-PV	D-PV	D-PV
VP (mbar)	50	50	50	20	20	20
FWC (wt.%)	15%	15%	50%	20%	25%	30%
MA (m^2^)	16	5	24	12	13	13
RE (wt.%)	99.5%	99.7%	99.5%	99.7%	99.7%	99.7%
EI (MWh/t)	0.8	1.6	0.9	1	1.2	1.4
LCOS (€/t)	177	276	190	181	197	215
EMI (t-CO_2_eq/t)	2.5	5.6	2.8	2.6	3.0	3.4
*COPCO* (€/t-CO_2_eq)	+24+	+3+	+22+	+23+	+21+	+19+

VP—vacuum pressure; FWC—feed water content; MA—membrane area; RE—recovery efficiency; EI—energy intensity; LCOS—levelized cost; EMI—emission intensity; *COPCO*—see Section 2.5.

## Data Availability

The original contributions presented in this study are included in the article/Supplementary Materials. Further inquiries can be directed to the corresponding author(s).

## Supplementary Materials

The following supporting information can be downloaded at: https://www.mdpi.com/article/10.3390/membranes15080224/s1, Table S1. Physicochemical properties of IPA and water; Table S2. VLE data for IPA–Water System at 1 atm; Table S3. NRTL parameters for IPA–water system; Table S4. Interpretation of the *COPCO* index; Table S5. Equipment cost data of the benchmark (D-D-D); Table S6. Equipment cost data of D-PV process; Table S7. Equipment cost data of D-PV-D process; Table S8. Equipment cost data of PV process; Table S9. Specifications of columns in azeotropic and hybrid configurations; Table S10. Technical results of all cases; Table S11. Economic results of all cases; Table S12. Environmental results of all cases; Table S13. Comparison with literature on IPA-water separation; Table S14. Significance analysis of key performance indicators; Table S15. Permeance and selectivity; Figure S1. LCOS breakdown at various vacuum pressures and feed water contents.
