# Supplementary material for: Unlocking the Benefits of Hybrid and Standalone Pervaporation for Sustainable Isopropanol Dehydration with HybSi® AR Membranes
Source: Membranes (Basel). 2025 Jul 26;15(8):224. doi: 10.3390/membranes15080224 (PMC12388116; doi:10.3390/membranes15080224)
Supplement: Supplementary file 1 [file membranes-15-00224-s001.zip › membranes-3741368-supplementary.pdf]

Supplementary Material

For manuscript

Unlocking the Benefits of Hybrid and Standalone Pervaporation for Sustainable Isopropanol Dehydration with HybSi® AR Membranes

Mohammed Nazeer Khan [1,*], Elmar Boorsma [2], Pieter Vandezande [1,*], Ilse Lammerink [2], Rob de Lange [2,3], Anita Buekenhoudt [1] and Miet Van Dael [1,4]

[1] Unit Materials & Chemistry (MatCh), Flemish Institute for Technological Research (VITO), Boeretang 200, 2400 Mol, Belgium.
[2] Pervatech B.V, Rijssen, The Netherlands.
[3] Aswyn, Engelse Schans 12, 7137 SE Lievelde, The Netherlands.
[4] Hasselt University, Centre for Environmental Sciences (CMK), Agoralaan, 3590 Diepenbeek, Belgium.

*Corresponding authors: pieter.vandezande@vito.be; mohammednazeer.khan@vito.be

S1. Annualized capital costs

The formula for the weighted average cost of capital (WACC) is described in equation [S1]. The WACC is the average cost of capital, taking into account the different sources of capital that a firm uses, and was estimated to be 4.8%. This value is based on equity ratio = 20%, debt ratio = 80%, discount rate = 9%, tax rate = 25% (Belgium), and interest rate = 5%.

$$WACC = (Equity * Discount\ rate) + \left(Debt * Interest\ rate * (1 - Tax\ rate)\right) \quad (S1)$$

The capital costs were annualized using the equation [S2].

$$Annualized\ cost = \frac{Total\ capital\ cost\ over\ plant\ lifetime}{\frac{1-(1+WACC)^{-Plant\ lifetime}}{WACC}} \quad (S2)$$

S2. Analytical method parameters

The water content during the pervaporation experiments is determined by coulometric Karl-Fischer (KF) titration for feed and retentate samples and volumetric titrations for permeate samples. For coulometric KF titration, an Aquacounter AQ-300 was used in combination with HYDRANAL – Coulomat A (Honeywell Fluka) as the anolyte and HYDRANAL – Coulomat CG (Honeywell Fluka) as the catholyte. For volumetric KF titration, a Metrohm 870 KF Titrino plus was used in combination with HYDRANAL Titrant 5 (Honeywell Fluka) as the titrating agent and HYDRANAL – Solvent (Honeywell Fluka) as the medium within the cell. The mass of the sample added to the KF cell was determined using a KERN precision balance ABT320-4M.

S3. Process modeling

Table S1. Physicochemical properties of IPA and water.

Property	Isopropanol (IPA)	Water
Molecular weight (g/mol)	60.1	18
Boiling point at 1 atm (°C)	82.4	100
Density at 25 °C (g/cm^3)	0.786	0.997
Viscosity at 25 °C (cP)	1.96	0.89
Enthalpy of Vaporization	44.0	40.7
Polarity index	3.9	10.2
Hydrogen bonding	Yes (donor and acceptor)	Yes (strong donor and acceptor)
Miscibility (with water)	Completely miscible	-

Table S2. VLE Data for IPA–Water System at 1 atm.

Boiling point	x_IPA	y_IPA
(°C)	(liquid mole fraction)	(vapor mole fraction)
82.05	1	1
81,02	0,9	0,8680806

80,42	0,8	0,7671075
80,19	0,7	0,6902029
80,25	0,6	0,6326707
80,5	0,5	0,5914686
80,84	0,4	0,5646454
81,13	0,3	0,5502463
81,35	0,2	0,5425692
82,51	0,1	0,5128721
100	0	0

Table S3. NRTL parameters for IPA-Water system.

Parameter	Value
Component i	C_3H_8O
Component j	H_2O
A_{ij}	-1.3115
A_{ji}	6.8284
B_{ij}	426.398
B_{ji}	-1483.46
C_{ij}	0.3
T_{lower}	25 °C
T_{upper}	100 °C

S4. *COPCO* index interpretation

Table S4. Interpretation of the *COPCO* index.

Scenario	Cost difference (€/t-IPA)	CO_2 difference (t-CO_2/t-IPA)	Cost per ton of CO_2 (€/t-CO_2)	Interpretation
1	positive	positive	+X+	€ saved per CO_2 saved
2	negative	positive	-X+	€ increased per CO_2 saved
3	positive	negative	+X-	€ saved per CO_2 emitted
4	negative	negative	-X-	€ increased per CO_2 emitted

S5. Economic data at VP = 20 mbar

Table S5. Equipment cost data of the benchmark (D-D-D).

Equipment	Capacity (kg/h)	Cost (€)
Column 1	1215	127,500
Column 2	1866	139,700
Condenser	1364	14,600
Decanter	1375	21,900
Column 3	225	66,600

Table S6. Equipment cost data of the D-PV process.

Equipment	Capacity (kg/h)	Cost (€)

Column 1	1,000	92,900
Pump	587	6,200
Heater	587	9,900
Pervaporation	587	227,466
Interstage heater	87	9,900
Condenser	87	10,900
Vacuum pump	1.3 kg/h·kPa	8,055
Chiller	64 kW	22,702

Table S7. Equipment cost data of the D-PV-D process.

Equipment	Capacity (kg/h)	Cost (€)
Column 1	1,000	100,700
Pump	960	6,000
Heater	960	11,300
Pervaporation	960	83,986
Interstage heater	86	11,200
Condenser	86	11,600
Vacuum pump	1.1 kg/h·kPa	7,767
Chiller	63.6 kW	22,649
Column 2	873.6	293,300

Table S8. Equipment cost data of the PV process.

Equipment	Capacity (kg/h)	Cost (€)
Pump	1,000	6,000
Heater	1,000	9,900
Pervaporation	1,000	405,884
Interstage heater	499	12,500
Condenser	499	16,600
Vacuum pump	1.3 kg/h·kPa	8,331
Chiller	369.6 kW	65,101

S6. Column specifications at VP = 20 mbar

Table S9. Specifications of columns in azeotropic and hybrid configurations.

Process		Column 1	Column 2	Column 3
D-D-D	Stages	10	20	10
	Feed stage	8	8	4
	Reflux ratio	2.48	0.96	0.52
D-PV	Stages	10	-	-
	Feed stage	8	-	-
	Reflux ratio	0.67	-	-
D-PV-D	Stages	10	28	-
	Feed stage	8	13	-
	Reflux ratio	0.67	3.8	-

S7. Technical results

Table S10. Technical results of all cases.

Process	Vacuum pressure	Feed water content	Product output	Recovery efficiency	Electricity	Steam	Cooling water
	mbar	wt.%	ton/yr	%	MWh/yr	ton/yr	m^3/yr
D-D-D	-	-	4,008	99.7%	0*	14,082	619,021
D-PV	20	15%	4,005	99.6%	164	5,914	173,323
D-PV-D	20	15%	4,010	99.8%	163	11,557	466,523
PV	20	50%	4,005	99.6%	884	6,059	421
D-PV	50	15%	4,001	99.5%	7	5,915	216,473
D-PV-D	50	15%	4.008	99.7%	7	11,554	509,165
PV	50	50%	4,000	99.5%	7	6,059	249,166
D-PV	20	20%	4,010	99.7%	228	6,831	201,439
D-PV	20	25%	4,007	99.7%	301	7,874	233,900
D-PV	20	30%	4,008	99.7%	384	9,062	270,970

**Electricity required for pumping was negligible and neglected*

S8. Economic results

Table S11. Economic results of all cases.

Process	Vacuum pressure	Feed water content	Capital costs	Operating costs	LCOS
	mbar	wt.%	€/yr	€/yr	€/t-IPA
D-D-D	-	-	186,552	931,188	279
D-PV	20	15%	175,221	474,710	162
D-PV-D	20	15%	263,075	835,940	274
PV	20	50%	223,530	470,634	173
D-PV	50	15%	219,103	489,893	177
D-PV-D	50	15%	265,279	839,236	276
PV	50	50%	256,306	502,861	190
D-PV	20	20%	196,351	529,165	181
D-PV	20	25%	206,886	583,934	197
D-PV	20	30%	216,689	645,502	215

S9. Environmental results

Table S12. Environmental results of all cases.

Process	Vacuum pressure	Feed water content	Annual emissions	Emission intensity	*COPCO* index
	mbar	wt.%	t-CO_2/yr	t-CO_2/t-IPA	€/t-CO_2
D-D-D	-	-	27,161	6.8	-
D-PV	20	15%	8,825	2.2	+25+
D-PV-D	20	15%	21,063	5.3	+3+
PV	20	50%	3,803	0.9	+18+
D-PV	50	15%	10,103	2.5	+24+
D-PV-D	50	15%	22,323	5.6	+3+
PV	50	50%	11,172	2.8	+22+
D-PV	20	20%	10,235	2.6	+23+

D-PV	20	25%	11,852	3.0	+21+
D-PV	20	30%	13,698	3.4	+19+

S10. LCOS comparison when VP = 20 mbar and 50 mbar

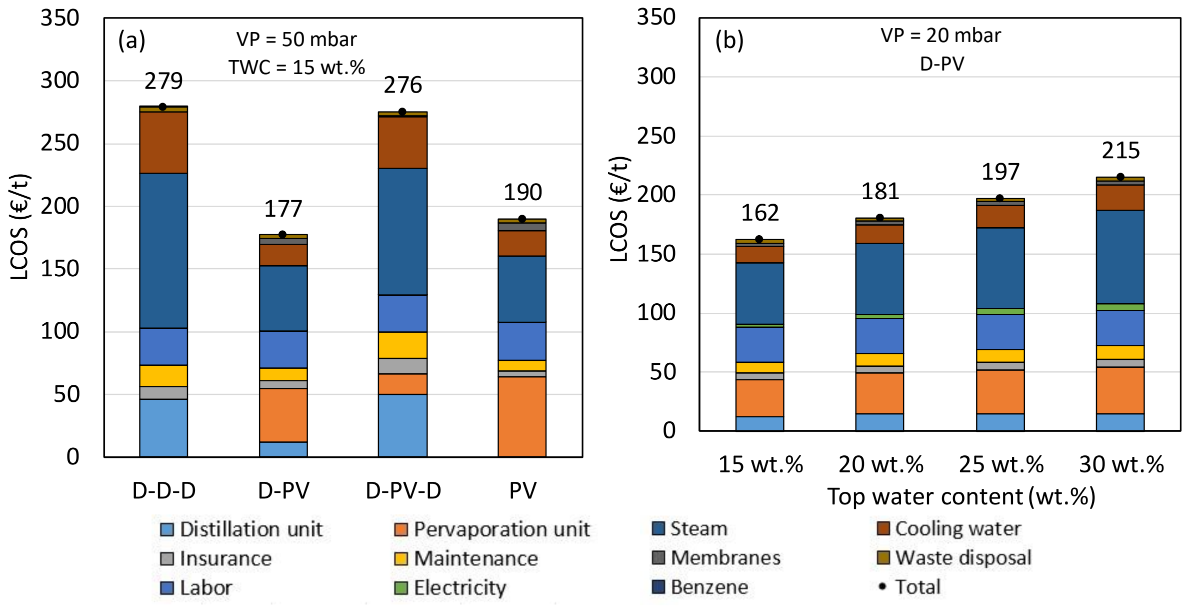

Figure. S1. LCOS breakdown at various vacuum pressures and feed water contents.

S11. Comparison with literature

Table S 13. Comparison with the literature on IPA-water separation.

Study	Process	Membrane type	Water flux (kg/m²·h)	Operating temp (°C)	LCOS (€/t-IPA)
This study (HybSi®)	Pervaporation (D-PV)	Hybrid silica (HybSi®)	~26*	130	173
Van Hoof et al. (2004)	Pervaporation (D-PV)	Zeolite NaA	~4*	95	66**
Harvianto et al. (2021)	Pervaporation (D-PV)	Zeolite NaA	~4*	95	70**
Harvianto et al. (2021)	Vapor permeation (D-VP)	P84 co-polyimide	0.3*	95	48**

**Water flux at 10 wt.% water feed*

***Values have not been updated to reflect current market conditions and are reported as-is.*

S12. Significance analysis

Significance analysis was performed by comparing key metrics (e.g., LCOS, CO_2 emissions) across configurations and calculating the maximum percentage differences to highlight meaningful performance gaps.

Table S14. Significance analysis of key performance indicators.

Metric	D-PV	D-PV-D	PV	Max. % difference
LCOS (€/t-IPA)	162	274	173	69.1%
Membrane area (m^2)	11	4	19	375%
Steam demand (t/t-IPA)	1.48	2.88	1.51	94.6%
CO_2 emissions (t-CO_2eq/t-IPA)	2.2	5.3	0.9	488.9%

S13. Permeance and selectivity

Permeance (Q_{H_2O}) was defined as the molar flux divided by the partial pressure difference across the membrane, while selectivity (or separation factor) was defined as the ratio of product water-to-IPA concentrations in the permeate and feed, respectively. These formulations are consistent with established definitions in pervaporation and membrane science [1–3].

Permeance and selectivity are challenging to calculate precisely due to the varying water content in the feed during dehydration. Since both parameters are strongly dependent on feed composition, particularly water concentration, a representative measurement point with similar water content was selected to ensure a meaningful and comparable calculation.

Table S15. Permeance and selectivity.

Experimental feed temperature (°C)	Vacuum pressure (mbar)	Water content feed (wt.%)	Selectivity (-)	Permeance (GPU)
115	20	3.5	977	3641
117	50	4.5	582	4243

The selectivity of the membrane, β, was calculated in the following way:

$$\beta = \frac{m^p_{H_2O}/m^p_{IPA}}{m^f_{H_2O}/m^f_{IPA}}$$

where $m^p$is the mass fraction in the permeate of water and IPA, and m^f is the mass fraction of water and IPA.

The permeance of the membrane for water, Q_{H_2O}, is calculated as the molar flux divided by the partial vapour pressure across the membrane in the following way:

$$Q_{H_2O} = \frac{J_{H_2O}}{\Delta p_{H_2O}}$$

where J_{H_2O} is the flux of water in kmol m^{-2} s^{-1} and Δp_{H_2O} is the partial vapour pressure of water across the membrane in kPa. The unit of permeance in the equation above is kmol m^{-2} s^{-1} kPa^{-1} can be rewritten in another commonly used unit, the gas permeation unit (GPU), where 1 kmol m^{-2} s^{-1} kPa^{-1} is equal to 2,99 x 10^9 GPU.

S14. Reference

1. Baker, R.W.; Wijmans, J.G.; Huang, Y. Permeability, Permeance and Selectivity: A Preferred Way of Reporting Pervaporation Performance Data. *J. Memb. Sci.* **2010**, *348*, 346–352, doi:10.1016/J.MEMSCI.2009.11.022.
2. Vane, L.M. Review of Pervaporation and Vapor Permeation Process Factors Affecting the

Removal of Water from Industrial Solvents. *J. Chem. Technol. Biotechnol.* **2020**, *95*, 495–512, doi:10.1002/jctb.6264.

3. Jyoti, G.; Keshav, A.; Anandkumar, J. Review on Pervaporation: Theory, Membrane Performance, and Application to Intensification of Esterification Reaction. *J. Eng. (United Kingdom)* **2015**, *2015*, doi:10.1155/2015/927068.
